# Human dental pulp stem cells transplantation combined with treadmill training in rats after traumatic spinal cord injury

**DOI:** 10.1590/1414-431X20165319

**Published:** 2016-08-08

**Authors:** F.C. Nicola, L.P. Rodrigues, T. Crestani, K. Quintiliano, E.F. Sanches, S. Willborn, D. Aristimunha, L. Boisserand, P. Pranke, C.A. Netto

**Affiliations:** 1 Programa de Pós-Graduação em Neurociências, Universidade Federal do Rio Grande do Sul, Porto Alegre, RS, Brasil; 2 Departamento de Bioquímica, Universidade Federal do Rio Grande do Sul, Porto Alegre, RS, Brasil; 3 Laboratório de Hematologia e Célula Tronco, Universidade Federal do Rio Grande do Sul, Porto Alegre, RS, Brasil; 4 Instituto de Pesquisas com Células Tronco, Porto Alegre, RS, Brasil

**Keywords:** Spinal cord injury, Stem cells, Treadmill training, Functional recovery, MASCIS impactor

## Abstract

Spinal cord injury (SCI) is a disabling condition resulting in deficits of sensory and motor functions, and has no effective treatment. Considering that protocols with stem cell transplantation and treadmill training have shown promising results, the present study evaluated the effectiveness of stem cells from human exfoliated deciduous teeth (SHEDs) transplantation combined with treadmill training in rats with experimental spinal cord injury. Fifty-four Wistar rats were spinalized using NYU impactor. The rats were randomly distributed into 5 groups: Sham (laminectomy with no SCI, n=10); SCI (laminectomy followed by SCI, n=12); SHEDs (SCI treated with SHEDs, n=11); TT (SCI treated with treadmill training, n=11); SHEDs+TT (SCI treated with SHEDs and treadmill training; n=10). Treatment with SHEDs alone or in combination with treadmill training promoted functional recovery, reaching scores of 15 and 14, respectively, in the BBB scale, being different from the SCI group, which reached 11. SHEDs treatment was able to reduce the cystic cavity area and glial scar, increase neurofilament. Treadmill training alone had no functional effectiveness or tissue effects. In a second experiment, the SHEDs transplantation reduced the TNF-α levels in the cord tissue measured 6 h after the injury. Contrary to our hypothesis, treadmill training either alone or in combination, caused no functional improvement. However, SHEDs showed to be neuroprotective, by the reduction of TNF-α levels, the cystic cavity and the glial scar associated with the improvement of motor function after SCI. These results provide evidence that grafted SHEDs might be an effective therapy to spinal cord lesions, with possible anti-inflammatory action.

## Introduction

Spinal cord injury (SCI) is a major neurological trauma with high rates of morbidity/mortality that affects people from all age groups (1). The lesion results in tissue loss, including myelinated tract fibers responsible for carrying sensory and motor information transmitted by descending and ascending pathways through the spinal cord (2). After the initial lesion, a chain of events take place leading to the disturbance of local circulation and the release of molecules that increase cell apoptosis near the injury site, such as TNF-α and other cytokines (3,4). Tissue loss leads to cystic cavity formation that is surrounded by a glial scar, composed mainly by reactive astrocytes, which contribute to motor impairment (3).

Injury severity and progression are key factors in gait recovery after SCI and directly impact the response to therapeutic agents. Treadmill training (5) and cell therapy (6,7) have been used to treat experimental SCI with promising results. Treadmill training promotes neuroplasticity induced by repeated locomotor activity (8) and axonal sprouting near the lesion site. It also increases synapses and decreases muscular atrophy, which are correlated with improved postural orientation and stability of paretic limbs (9).

Stem cells (SCs) are primitive undifferentiated and unspecialized cells with self-renewal capacity (10), and their transplantation has shown encouraging results in SCI treatment. Recently, bone marrow stem cells, neural stem/progenitor cells, umbilical cord blood stem cells and stem cells from human exfoliated deciduous teeth (SHEDs) have been transplanted after experimental SCI and have promoted motor function recovery (6,11,12).

SHEDs are considered a valuable source of stem/progenitor cells. These cells are found in the dental pulp perivascular niche and have well documented self-renewal ability (13). The collection of SHEDs is simple and its use presents no ethical concerns. Some of the advantages of these cells, compared to other stem cells, are: the dental pulp can be easily dissected and cells can be stored for long terms in liquid nitrogen to be further used without immunological risks (14); and SHEDs show greater cell proliferation rate when immediately transplanted after SCI-induced functional recovery (12).

Due to the recovery potential shown by SHEDs transplantation and by treadmill training following experimental SCI, we investigated whether the association of both treatments would produce greater motor function recovery after traumatic SCI in Wistar rats. The working hypothesis was that the combined treatment would produce greater improvement, or accelerate the functional recovery after SCI, when compared with rats treated only with SHEDs or the motor training. Additionally, a possible anti-inflammatory action of the neuroprotective treatments was assayed with the measurement of TNF-α levels in the cord tissue.

## Material and Methods

### Isolation of SHEDs and cell culture

The collection, isolation and culture of stem cells from human exfoliated deciduous teeth were accomplished according to Bernardi et al. (15). The donor gave written informed consent to participate in the study, which was approved by the Ethics Committee of the Universidade Federal do Rio Grande do Sul (#296/08) (15).

Briefly, the dental pulp was removed and incubated at 37°C for 60 min in buffer containing 0.2% type 1 collagenase (Gibco, USA). Cells were disrupted from the dental pulp and cultured, according to Luisi (16). All pulp tissue was removed (crown and root) from the dentin and the resulting cell suspension was seeded onto a 12-well plate. The culture medium DMEM (Dulbecco's), supplemented with 10% fetal bovine serum (Laborclin, Brazil), was changed 24 h after the initial plating and, after that, every 3 or 4 days. When the culture reached 90% confluence, a passage using trypsin-EDTA 0.5% (Sigma-Aldrich, USA) was performed to loosen the cells from the plate. The density of cells seeded in each passage was 10^4^ cells/cm^2^. Cells of the 5th passage were utilized for cell transplantation and for culture characterization analysis. The cells were incubated with bis-benzimide (Hoechst 33342) (5 µg/mL, Sigma, USA) for 5 min and washed three times with PBS before transplantation.

### Flow cytometry analysis

Flow cytometry analysis was performed in cultures from human exfoliated teeth in the 5th passage (n=3) (15). 10^6^ cells were incubated with the following conjugated antibodies against human cell surface molecules: CD29, CD34 (hematopoietic stem/progenitor cells/endothelium), CD44, CD45, CD73, CD90 (common leukocyte antigens), HLA-DR (human leukocyte antigen, class II), CD14 (monocyte/macrophage), CD184 and STRO-1 (Stromal Cell Surface Marker) (PharMingen-BD Biosciences, USA), conjugated with FITC (Santacruz, USA) or PE (PharMingen-BD Biosciences). Data acquisition was performed using the FACSAria III flow cytometer (BD Biosciences, USA) and 10,000 events were analyzed using the FACS Diva 6.1.3 software (BD Biosciences).

### First experiment

**Figure 1:**
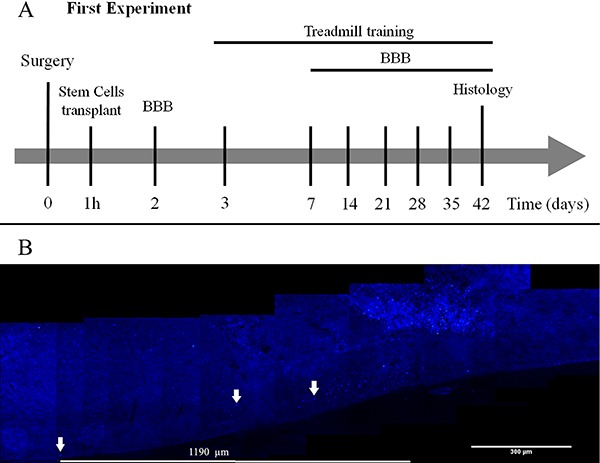
Experimental design (*A*). Photomicrographs of stem cells from human exfoliated deciduous teeth (SHEDs) labeled with Hoechst 33342 (*B*). SHEDs survived and migrated 1.190 µm along the spinal cord (arrows). BBB: Basso, Beatie, and Bresnahan scale.

Fifty-four adult male Wistar rats aged 2 months (200-250 g body weight) were obtained from the Animal House of the Instituto de Ciências Básicas da Saúde of the Universidade Federal do Rio Grande do Sul. They were maintained in a temperature-controlled room (21±2°C) on a 12/12 h light/dark cycle, with food and water available *ad libitum*. All procedures were in accordance with the Guide for the Care and Use of Laboratory Animals adopted by the National Institute of Health (USA) and with the Federation of Brazilian Societies for Experimental Biology. The study was approved by the Research Ethics Committee of the University (#22249). Animals were randomly divided into five experimental groups: surgical control, Sham (laminectomy with no SCI, n=10); SCI (laminectomy followed by SCI, n=12); SHEDs (SCI treated with SHEDs, n=11); TT (SCI treated with treadmill training, n=11); SHEDs+TT (SCI treated with SHEDs and treadmill training, n=10). There was a 10% death rate after the surgical procedure. Animal care was in accordance with the Multicenter Animal Spinal Cord Injury Study (MASCIS) protocols (17). The experimental design can be visualized in Figure 1A.

### Spinal cord injury and SHEDs transplant

SCI was performed using the New York University Impactor (NYU-Impactor^®^; W.M. Keck Center for Collaborative Neuroscience, USA). Animals were previously anesthetized with a mixture of xylasine (100-150 mg/kg) and ketamine (60-90 mg/kg). Laminectomy was performed at thoracic vertebral level 9 (T9), and injury was induced through the drop of a 10 g weight from 25 mm height. Animals transplanted with SHEDs received 3×10^5^ cells diluted in 0.9% NaCl. A 10-µL cell suspension was injected at the lesion site 1 h after the injury with a 25-µL sterile Hamilton syringe, and carried out without immunosuppression (18). Following the SCI procedure, animals were sutured and housed in individual cages and bladder evacuation was performed daily until they recover the function. Antibiotic (Enrofloxacino, Bayer, Brazil; 6 mg/kg) was administered for 7 days after the procedure to prevent infection. In order to show the presence of transplanted SHEDs into the cord tissue, cells were labeled with Hoechst 33342 and injected at the lesion site of a group of spinalized rats.

### Treadmill training

One week before the surgical procedure, the animals were randomly assigned to the treadmill training. First, rats were adapted to the treadmill (Insight^®^, Brazil) with 20-min sessions, once a day for 5 days, running at a constant speed of 10.5 m/min. TT and SHEDs+TT groups began training 3 days after surgery: one session per day with treadmill speed of 10.5 m/min, 5 days per week for 6 weeks (19). No body weight support was offered to the groups.

### Locomotor activity assessment

The functional recovery was evaluated during spontaneous locomotor activity in the open field by the use of the Basso, Beatie, and Bresnahan scale (BBB), that assesses hind limb motor function with scores ranging from 0 (complete paralysis) to 21 (normal locomotion) (20). Evaluation started 2 days before the injury or laminectomy (in the Sham group), was repeated 2 days after SCI and then once a week for 6 weeks. Locomotor activity in the open field was videotaped and observed for scale scoring by two examiners who were blind to the animal's experimental group (20); all animals were assessed.

### Morphological analysis

After completing behavioral assessment, i.e., 6 weeks after surgery, rats were anesthetized with pentobarbital (100 mg/kg *ip*; Cristália, Brazil) and submitted to transcardiac perfusion with 0.9% saline followed by 4% paraformaldehyde (Reagen, Brazil) in 0.1 M phosphate buffer (PBS, pH 7.4). After this procedure, the spinal cord was removed, from C5 to L5 in the thoracic region, post-fixed in the same fixative solution and cryoprotected with 15 and 30% sucrose diluted in phosphate buffer saline (PBS). After cryoprotection, samples were frozen in isopentane, cooled in liquid nitrogen until slicing (21). For histological and immunofluorescence analysis, the thoracic region of the spinal cord was transversely cut (5,6) into 20 µm sections in cryostat (Leica, Germany). The sections were stained with hematoxylin and eosin and the images were captured using a Nikon Eclipse E-600 microscope (Japan) coupled with a digital camera.

Thirty transversal 20-µm sections from each animal were processed to analyze the cavitation area. Sequential sections with an interval of 300 µm were collected. Cavitation area in each sequential slice was determined and the largest cavitation area slice (called the epicenter) of each rat was determined (19). The cavitation area was traced using the software Image J v. 1.46 (http://rsbweb.nih.gov/ij/); any necrotic tissue within the cavities was considered as part of the lesion and the total sum of areas was determined (11).

### Immunofluorescence

Sectioned slices were washed with PBS, and membranes were permeabilized in 0.25% PBS-Triton X. Sections were then blocked with 1% albumin for 30 min. Primary antibodies used were: antibody against glial fibrillary acidic protein (anti-GFAP, rabbit IgG, 1:200, Sigma-Aldrich) to identify astrocytes; against myelin basic protein (anti-MBP, rabbit, 1:100, Abcam) to identify oligodendrocytes, and against neurofilament medium (anti-NF-M, rabbit IgG, 1:500 AbD Serotec, UK) to identify neurons. This procedure was carried out in 1% albumin in PBS-Tx at 4°C for 24 h. Following PBS washes, sections were incubated with secondary antibody anti-mouse Alexa 488 (1:500, Molecular Probes, Invitrogen, USA) and secondary antibody anti-rabbit Alexa 555 (1:500, Molecular Probes, Invitrogen). Slices were covered in aqueous mounting medium (FluorSave, Calbiochem, Germany) and coverslipped.

### Quantitative image analysis

GFAP, MBP and NF-M staining intensities were assessed in transversal slices containing the larger cavity area (epicenter) as well as one slice above and one below the epicenter (3 slices per animal). GFAP intensity was quantified around the lesion site, and MBP and NF-M at the ventral white matter (VWM); all analyses were made using high magnification images (20×). An area of interest (AOI) was determined (3.800 μm^2^) to assess the staining intensity. Two AOIs of each slice around the spinal cord cavity or VWM were obtained from 5 animals per group.

The captured images were analyzed using the software Image J v. 1.46 and the integrated density value per unit of area was obtained. Data are reported as the mean integrated densities/μm^2^, as previously described by Jeong et al. (22).

### Second experiment

**Figure 2:**
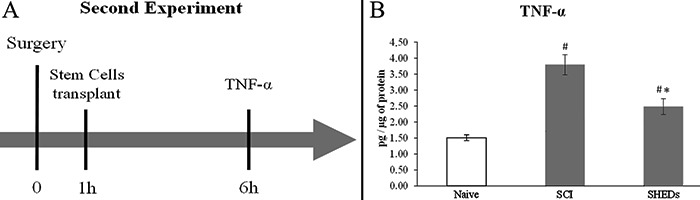
Design of the second experiment (*A*). ELISA results for TNF-α (*B*). Naive: rats with no manipulation; SCI: rats with laminectomy followed by spinal cord injury; and SHEDs: rats with SCI treated with stem cells from human exfoliated deciduous teeth (SHEDs). Data reported as means±SE. ^#^P<0.05, compared to Naive group; *P<0.05, compared to SCI group (one-way ANOVA followed by Tukey's *post hoc* test).

In order to assess a possible role of inflammation in SHEDs mechanism of action, fourteen adult male Wistar rats aged 2 months (200-250 g body weight) were randomly divided into three experimental groups: Naive control (animals with no manipulation); SCI (laminectomy followed by SCI) and SHEDs (SCI treated with SHEDs). Six hours after the procedure rats were anesthetized with pentobarbital (100 mg/kg, *ip*; Cristália) and beheaded, and spinal cords were then removed to proceed with the ELISA assay (n= 4-6) (Figure 2A).

### TNF-α assay

TNF-α concentration in the spinal cord tissue was measured by using TNF-α ELISA kit (eBioscience, Ref. 88-7340, USA). Spinal cord tissue at the epicenter was collected and prepared according to the manufacturer's protocol. Protein was estimated by the commercial kit BCA Protein Assay (Thermo Scientific, USA, Ref. #23225), to allow the estimation of cytokine concentration.

### Statistical analysis

Data are reported as means±SE. Statistical analysis of motor behavior (BBB score) was performed using repeated-measures analysis of variance (ANOVA) to detect treatment effects over time. Individual ANOVAs were performed to compare intra-day differences in BBB scores among groups (23). One-way ANOVA was used to analyze quantitative image data, followed by Tukey's *post hoc* test to identify differences between groups whenever indicated. Significance was assumed at P<0.05.

## Results

*Characterization of isolated SHEDs*. Flow cytometry analysis showed that SHEDs expressed a set of mesenchymal stem cell (MSC) markers (CD44, CD73, CD90, CD29), and no hematopoietic/endothelial markers (CD14, CD34, CD45, CD184, HLA-DR, STRO-1) (Table 1). The differentiation into adipogenic, condrogenic and osteogenic cells (data not shown) was observed, as previously reported (15).

**Table:**
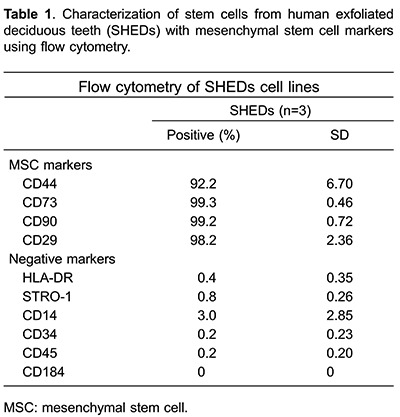

*Cell survival.* Three animals received SHEDs labeled with Hoechst 33342 to identify their presence and migration into the spinal cord 42 days post-injury. The labeled cells were observed in the spinal cord tissue reaching a distance of 1.190 µm from the lesion epicenter (Figure 1B).

**Figure 3:**
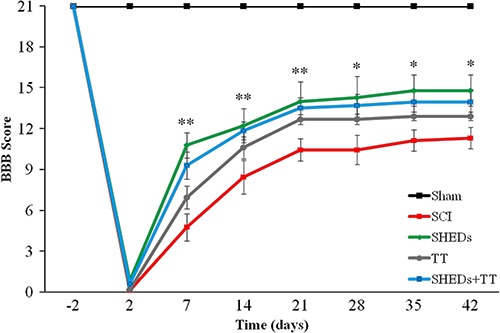
Progress of hind limbs functional recovery after incomplete spinal cord injury (SCI), as evaluated with the BBB scale, in sham operated (Sham), SCI, SCI treated with stem cells from human exfoliated deciduous teeth (SHEDs), SCI treated with treadmill training (TT), and SCI treated with SHEDs and treadmill training (SHEDs+TT) rats. Data are reported as means±SE. *Significantly different comparing the SHEDs group to the SCI group; **significantly different comparing the SHEDs and the SHEDs+TT groups to the SCI group. Statistical analysis was performed using repeated-measures ANOVA to detect treatment effects over time, and individual one-way ANOVA followed by Tukey's *post hoc* were performed to observe intraday differences among groups (P<0.05). BBB: Basso, Beatie, and Bresnahan scale.

*SHEDs improved locomotor recovery after SCI*. Repeated measures ANOVA showed a group effect over the weeks in functional recovery. SHEDs group exhibited an increase in BBB scores, as compared to SCI group, from the first to the sixth week. A similar effect was observed in SHEDs+TT as compared to SCI group, from the first to the third week, however the TT group was not distinct from the SCI group, showing no recovery after training; the Sham group had no locomotor impairment, as expected. The Sham group had maximum BBB score (21±0.0) observed in the last experimental week, being different from SCI (11±0.76), SHEDs (15±1.1), TT (13±0.32), and SHEDs+TT (14±0.93) groups (F(4.46)=65.31; P<0.001; Figure 3). These results demonstrate that SHEDs transplantation during the acute phase of injury improved recovery of hind limb function.

**Figure 4:**
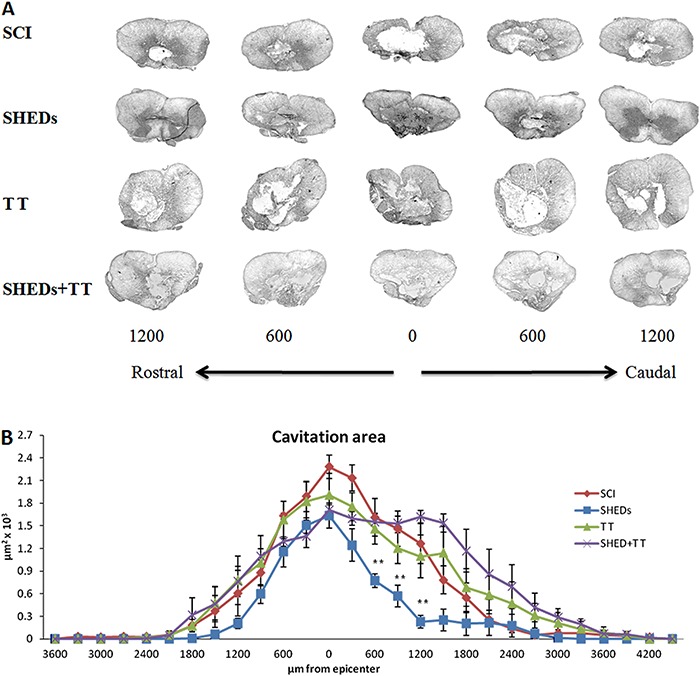
Representative images of cavitation areas (*A*) and cavitation area analysis (*B*) of incomplete spinal cord injury (SCI), SCI treated with stem cells from human exfoliated deciduous teeth (SHEDs), SCI treated with treadmill training (TT), and SCI treated with SHEDs and treadmill training (SHEDs+TT) rats. Data are reported as means±SE. **P<0.05, SHEDs group compared to SCI group (one-way ANOVA followed by Tukey's *post hoc* test).

*SHEDs transplantation improved tissue preservation after SCI*. The cystic cavity areas, as well as the expression of glial-scar marker (GFAP), were decreased in the SHEDs group when compared to the SCI group. SHEDs transplantation decreased cavity area at 600 µm (0.77±0.09/μm^2^) and 1200 µm (0.23±0.08/μm^2^) caudally to the lesion epicenter (1.63±0.16/μm^2^), when compared to the SCI group. However, all injured groups presented similar damage at the epicenter. The mean cavity areas observed were 2.28±0.15/μm^2^ in the SCI group, 1.63±0.16/μm^2^ in the SHEDs group, 1.90±0.29/μm^2^ in the TT group and 1.70±0.07/μm^2^ in the SHEDs+TT group (Figure 4A and B).

**Figure 5:**
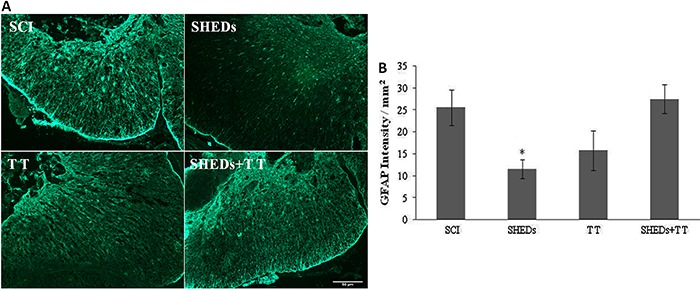
Representative immunostaining of GFAP expression in the epicenter of injured spinal cord, 6 weeks after spinal cord injury (SCI), SCI treated with stem cells from human exfoliated deciduous teeth (SHEDs), SCI treated with treadmill training (TT), and SCI treated with SHEDs and treadmill training (SHEDs+TT) rats (*A*). Densitometry of GFAP (*B*). Data are reported as means±SE. *P<0.05, SHEDs group compared to SCI group (one-way ANOVA followed by Tukey's *post hoc* test). Scale bar = 50 µm.

*SHEDs caused a decrease in glial scar formation along the preserved tissue* (Figure 5A). Animals that received only SHEDs transplantation presented a decrease of GFAP expression at the lesion site (11.47±2.14/mm^2^), as compared to the SCI group (25.47±4.03/mm^2^). GFAP expression at the lesion site was increased in TT rats (15.71±4.51/mm^2^), while in the SHEDs+TT, expression (27.42±3.28/mm^2^) was similar to that of the SCI group, (F(3.16)=4.52; P<0.018) (Figure 5B).

**Figure 6:**
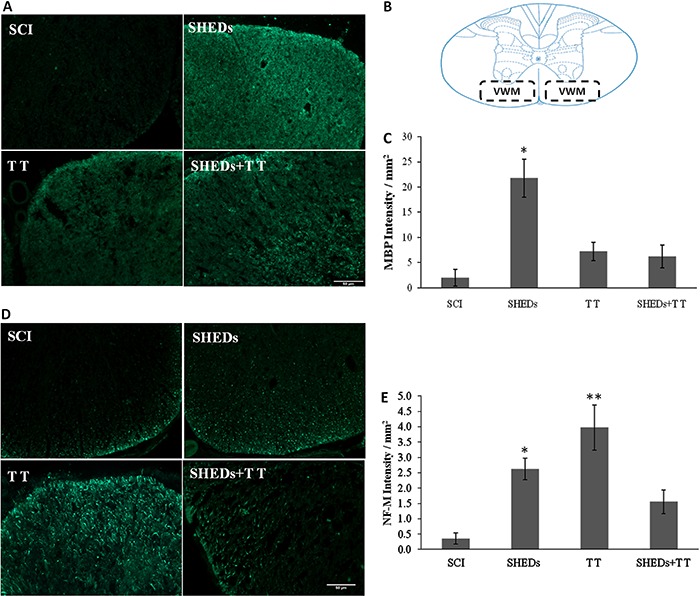
MBP staining in ventral white matter (VWM) (*A*), spinal regions of quantification (*B*), densitometry of MBP (*C*), spinal regions of NF-M staining in VWM (*D*), densitometry of NF-M (*E*) of spinal cord injury (SCI), SCI treated with stem cells from human exfoliated deciduous teeth (SHEDs), SCI treated with treadmill training (TT), and SCI treated with SHEDs and treadmill training (SHEDs+TT) rats. Data are reported as means±SE. *P<0.05, SHEDs group compared to SCI group. **P<0.05, TT group compared to SHEDs and SCI groups (one-way ANOVA followed by Tukey's *post hoc* test). Scale bar = 50 µm.

*SHEDs promoted MBP and NF-M preservation.* Forty-two days after the lesion, myelin was evaluated at the ventral white matter (Figure 6B). The SHEDs group showed an increase of MBP expression (21.71±3.77/mm^2^), being different from the SCI group (1.95±1.65/mm^2^). Both TT (7.20±1.77/mm^2^) and SHEDs+TT (6.21±2.28/mm^2^) groups did not differ from the SCI group (F(3.16)=11.68; P<0.001; Figure 6A-C). Similarly, axonal fibers were evaluated by NF-M at the VWM (Figure 6B). The SHEDs group had greater NF-M expression at the lesion epicenter (2.60±0.35/mm^2^) indicating axonal fiber preservation, as compared to the SCI group (0.35±0.18/mm^2^). The TT group showed higher NF-M expression (3.97±0.73/mm^2^) when compared to the SCI and the SHEDs+TT (1.60±0.38/mm^2^) groups. SHEDs+TT did not differ from the SCI group (F(3.16)=11.09; P<0.001) (Figure 6D and E).

*SHEDs transplant reduced TNF-α levels.* In order to reveal a possible anti-inflammatory action of transplanted SHEDs, an experiment to measure the expression of TNF-α was conducted. Six hours after the spinal cord injury TNF-α expression was elevated in the SCI group (3.80±0.31 pg/ug of protein) when compared to Naive (1.51±0.09 pg/ug of protein); conversely, the SHEDs group (2.49±0.25 pg/ug of protein) showed a reduction of TNF-α when compared to the SCI group (Figure 2B). This result suggests that transplanted cells prevented the overexpression of TNF-α after the spinal lesion through an anti-inflammatory action.

## Discussion

This is the first study investigating the use of stem cells derived from human exfoliated deciduous teeth in combination with treadmill training, to treat experimental spinal cord injury. Results demonstrate that SHEDs treatment promoted functional recovery, decreased the cystic cavity and glial scar, and increased neurofilament density near the lesion site after traumatic SCI. Surprisingly, the treadmill training did not promote functional recovery after SCI, neither alone nor in combination with SHEDs.

In agreement with previous findings, SHEDs transplantation one hour after the injury promotes functional improvement assessed by the BBB scale (12,24). In the present study SHEDs produced better performance since the 1st week after SCI, an effect maintained until the 6th week post-injury. The improvement observed in the early phase of functional recovery confirms the potential of SHEDs and suggests a neuroprotective, instead of a neuroregenerative action (12,24). Over the weeks, the SHEDs group exhibited greater BBB scores when compared to SCI group, reaching 3.9 points of difference.

### Treadmill training

Contrary to our working hypothesis, treadmill training starting 3 days after SCI (19) did not improve functional recovery when performed alone (Figure 2). The protocol of treadmill training here employed can be classified as an aerobic exercise, once it involves large muscle groups in dynamic activities that result in substantial increases in heart rate and energy expenditure (25). The literature already shows controversial results in motor function when treadmill training is applied after experimental SCI, suggesting that the time-window and intensity of training affect the recovery protection of motor training (26).

Exercise up-regulated brain-derived neurotrophic factor (BDNF) in rats trained from 14 to 20 days after SCI, however this effect was not observed when animals were trained from 0 to 6 days post-injury (27). It seems that treadmill training starting at early phases (acute periods), in the presence of excitatory and toxic substances released from the injured tissue, might have detrimental effects by the additional release of these substances by locomotor activity (28). Another study (29) showed that treadmill training failed to enhance motor function when started at the early phase of recovery after SCI, an effect associated with similar sparing in the white matter between trained and untrained rats. Battistuzzo et al. (26) have shown positive results using treadmill training protocols, despite controversies. Nevertheless, additional studies are required to define the conditions in which treadmill training might have clear positive effects in spinalized rats.

Another point to be considered is that, although being the current method for the assessment of locomotor ability after SCI (29), the BBB scale presents limitations that might mask improvements after a certain level (30). An important disadvantage is that its ordinal rating system is not linear: the lower part of the scale, from 0 to 12 points, relates to gross aspects of locomotion; while the upper part of the scale, 13 to 21 points, includes rather discrete movement aspects that do not represent major improvements for the animal's motor ability. The difference between animals is very subtle, especially in animals with high locomotor outcome and stable performance (BBB score >13), such as the treated groups in the present study.

### Tissue preservation after SCI

Reactive astrogliosis occurs after injury to the central nervous system as a result of glial scar formation following tissue loss and cavitation establishment. Evaluation of the cavitation area is a common method used to assess spinal cord lesion size (6,19,22), and the present data showed that SHEDs transplantation reduced cavity area (Figure 4); surprisingly, treadmill training did not promote tissue preservation, i.e., TT and SHEDs+TT groups had larger cavitation areas in the 6th week (Figure 4). Tissue preservation near the injury site would possibly preserve specialized cells, such as oligodendrocytes, that directly improve motor function (12,24); however, the exact mechanism by which SHEDs produce such improvement is unknown.

Glial scar and cavitations are obstacles to axonal growth after injury, and inhibitors of glial scar have been used to promote axonal growth and locomotor improvement (31). A good marker of lesion extension and severity is the astrocyte response to injury (22), easily identified by GFAP immunofluorescence (6,12,19,26). Treatment with SHEDs reduced GFAP around the lesion site, in comparison to the SCI group, showing its potential to reduce the glial scar (Figure 5). This is in agreement with findings by Jeong et al. (22) that showed a decrease in astrocytic scar formation after mesenchymal stem cells transplant in SCI rats. Conversely, treadmill training caused an increase in GFAP expression around the lesion site, i.e., it was not able to reduce the glial scar in the anterior horn of the spinal cord, nor in the surrounding cavity lesion, as previously reported (32).

### SHEDs prevented demyelination and axonal loss

Spinal cord lesion selectively eliminates large axonal fibers and causes sustained demyelination (33). SHEDs transplantation prevented demyelination and probably preserved local neuronal viability, an effect that has been previously shown by other stem cell types transplanted to SCI rats (12). Treadmill training, either alone or in combination with SHEDs, did not prevented demyelination, an effect probably caused by the glial scar. In fact, glial scar has been associated with demyelination, thus hindering remyelination and axonal growth (3).

NF-M, a cytoskeletal axon constituent, is used to assess axonal preservation and has been correlated with functional recovery (9). Grafted SHEDs preserved NF-M expression in spinal cord, visualized in the 6th week after SCI, as previously reported (12). Conversely, the NF-M increased expression in the TT group was not associated with motor improvement. It is possible that aberrant axons that could emerge from the lesion site after SCI (34) would have physiological consequences, and potentially contribute to abnormal responses. The regular treadmill training may have induced a series of adaptive responses, which may have contributed to axonal disorder (35). It is believed that forced exercises, like treadmill training, that significantly increase cardiovascular output at acute or sub-acute post-SCI time-points might cause increased extravasations of macromolecules to the spinal cord parenchyma, which can alter secondary injury progression and impair recovery (36).

Although the mechanisms of action of SHEDs for promoting tissue protection and functional recovery remain under scrutiny, MSCs have already been used in clinical trials to assist in immunity re-establishment (37,38). Considering the evidence that SHEDs might exert neuroprotection through immune regulation (37), results from the second experiment demonstrate that SHEDs transplantation reduced TNF-α overexpression caused by SCI. TNF-α is a pro-inflammatory cytokine that induces apoptosis and is highly expressed after spinal cord injury, mainly in the first hours (4). This suggests that SHEDs neuroprotection was elicited, at least in part, by an anti-inflammatory action.

The fact that the combination between treadmill training and stem cells transplantation after SCI did not improve functional recovery in SCI rats implies that caution is needed in defining motor training protocols. Additional studies are needed in order to establish the adequate time-window and intensity of treadmill running to better explore its therapeutic potential. Confirming the working hypothesis, grafted SHEDs survived and integrated into the spinal cord tissue, reduced tissue damage and glial scar formation, and promoted functional recovery, probably due to an anti-inflammatory action. Thus, SHEDs transplantation constitutes a promising approach to treat spinal cord lesions.
